# The Effectiveness of Mobile Phone-Based Care for Weight Control in Metabolic Syndrome Patients: Randomized Controlled Trial

**DOI:** 10.2196/mhealth.4222

**Published:** 2015-08-20

**Authors:** Bumjo Oh, Belong Cho, Min Kyu Han, Hochun Choi, Mi Na Lee, Hee-Cheol Kang, Chang Hee Lee, Heeseong Yun, Youngho Kim

**Affiliations:** ^1^SMG-SNU Boramae Medical CenterDepartment of Family Medicine & Center for Health PromotionSeoulRepublic Of Korea; ^2^Seoul National University HospitalDepartment of Family MedicineSeoulRepublic Of Korea; ^3^Advanced Institutes of Convergence TechnologySeoul National UniversitySuwon–siRepublic Of Korea; ^4^Institute on AgingSeoul National University College of MedicineSeoulRepublic Of Korea; ^5^Severance HospitalDepartment of Family MedicineSeoulRepublic Of Korea; ^6^LG ElectronicsFuture IT R&D LabSeoulRepublic Of Korea

**Keywords:** metabolic syndrome X, obesity, diet, exercise, mobile health, health information management

## Abstract

**Background:**

Overweight and obesity, due to a Westernized diet and lack of exercise, are serious global problems that negatively affect not only personal health, but national economies as well. To solve these problems, preventative-based approaches should be taken rather than medical treatments after the occurrence of disease. The improvement of individual life habits, through continuous care, is thus a paramount, long-term treatment goal. This study describes the effects of ubiquitous health care (uHealth care) or SmartCare services in the treatment of weight loss and obesity.

**Objective:**

The aim of this study is to evaluate the effect of SmartCare services on weight loss compared to the effects of existing outpatient treatments in obese patients with metabolic syndrome.

**Methods:**

Metabolic syndrome patients who met the inclusion/exclusion criteria were enrolled in the study and randomized into an intervention or control group. The intervention group was provided with remote monitoring and health care services in addition to the existing treatment. The control group was provided with only the existing treatment. Pedometers were given to all of the patients. Additionally, mobile phones and body composition monitors were provided to the intervention group while body weight scales were provided to the control group. The patients visited the hospitals at 12 and 24 weeks following the baseline examination to receive efficacy and safety evaluations.

**Results:**

Mean weight reduction from baseline to week 24 was measured as a primary efficacy evaluation parameter and was found to be 2.21 kg (SD 3.60) and 0.77 kg (SD 2.77) in the intervention and control group, respectively. The intervention group had a larger decrement compared to the control group (*P*<.001). Among the secondary efficacy evaluation parameters, body mass index (BMI) (*P*<.001), body fat rate (*P*=.001), decrement of waist measurement (*P*<.001), and diet habit (*P*=.012) improvement ratings from baseline to week 24 were found to be superior in the intervention group compared with the control group. The proportion of patients whose body weight decreased by ≥10%, lipid profiles, blood pressure, prevalence of metabolic syndrome, change in the number of metabolic syndrome elements, smoking rate, drinking rate, and physical activity were not statistically significant between the groups.

**Conclusions:**

The efficacy of SmartCare services was confirmed as the intervention group that received both SmartCare services and the existing treatment had superior results compared with the control group that only received the existing treatment. Importantly, no specific problems with respect to safety concerns were observed. SmartCare service is thus an effective way to control the weight of obese patients with metabolic syndrome.

**Trial Registration:**

Clinicaltrials.gov NCT01344811; https://clinicaltrials.gov/ct2/show/NCT01344811 (Archived by Webcite at http://www.webcitation.org/6alT2MmIB)

## Introduction

Obesity increases the prevalence of diabetes mellitus type 2, metabolic syndrome, cardiovascular diseases, and the mortality rate [1]. Metabolic syndrome is a condition in which various cardiovascular and metabolic risk factors are simultaneously present because of insulin resistance, obesity, and other factors, causing cardiovascular diseases, the main causes of death [2,3]. The specific cause of metabolic syndrome is unknown, but it is important to control body weight because obesity and insulin resistance are suspected to be fundamental causes [4,5]. Furthermore, a substantial amount of research has reported that the occurrence of diabetes or macroangiopathy, a complication of diabetes, can be reduced by actively losing weight at the beginning of the disease, reinforcing the importance of reducing body weight and body fat through life habit improvements [2]. Obesity has been reported as the most important factor associated with metabolic syndrome. Therefore, it is necessary to recognize obesity as an individual disease and to treat it as such [6]. In 2014, the World Health Organization (WHO) reported that more than 1.9 billion adults, 18 years and older, were overweight. Of those, over 600 million were obese. Worldwide, the proportion of adults with a body mass index (BMI) of ≥25 kg/m^2^ increased between 1980 and 2013 from 28.8% to 36.9% in men, and from 29.8% to 38.0% in women [7]. According to the Korea National Health and Nutrition Examination Survey (KNHANES), a domestic epidemiologic survey performed by the Ministry of Health and Welfare, national obesity prevalence increased from 26.9% in 1998 to 32.0% in 2011 [8].

With the population aging and standards of living increasing, interest in personal health is on the rise. With respect to the advent of a ubiquitous era of advanced information technology, the field of ubiquitous health (uHealth) care, where information technology is combined with medical technology, is considered to be the new high-value industry of the future. uHealth care refers to the medical service that provides disease prevention, diagnosis, treatment, and care anytime and anywhere without physically visiting a hospital. In contrast to current medical concepts, which emphasize care and treatment after the onset of disease, uHealth care, along with the advancement of modern medicine, has the potential to discover and treat diseases in their early phases through pre-diagnosis and prevention [9]. Thus, uHealth care is developing into a broad concept with long-term sustainability for healthy living due to the improved quality and efficiency of medical services.

To prevent and control obesity, an exercise and diet intervention is necessary. Among a number of intervention strategies, a comprehensive body weight control strategy conducted on entire populations is effective in reducing medical costs and the economic burden of obesity [5,10-13]. Attempts to manage exercise and diet intake, the two key goals for weight loss, in real-time have been performed with limited means through phone, email, and short message service (SMS) text messaging [14-16]. However, as mobile phones became more prevalent and apps with concepts from uHealth care were introduced, studies were conducted that continued to develop and evaluate the efficacy of mobile phone-based apps [17-20].

This clinical trial was planned as a multicenter, randomized, parallel, and open-label study to evaluate the effect of uHealth care service (hereinafter referred to as SmartCare) on weight loss in obese patients with metabolic syndrome. A 24-week randomized controlled trial was conducted to determine whether SmartCare would be more effective in treating metabolic syndrome compared with the standard care in the hospital.

## Methods

### Recruitment

Of the male and female subjects aged ≥20 who visited one of the two general hospitals (Seoul National University Hospital and Severance Hospital) in Seoul, obese patients with metabolic syndrome were recruited. Those whose BMI was ≥25 kg/m^2^and who met at least 3 of the 5 following requirements were defined to have metabolic syndrome and were recruited as a subject in this trial.

According to the Adult Treatment Panel (ATP) III criteria using waist circumference cut-off modifications for Asian populations as suggested by the Asia-Pacific guidelines[21], metabolic syndrome is defined as having at least 3 of the following factors (1) central obesity (waist circumference ≥90 cm in men and ≥80 cm in women), (2) hypertriglyceridemia (triglyceride (TG) ≥150 mg/dL), (3) high-density lipoprotein cholesterol (HDL-C) <40 mg/dL in men and <50 mg/dL in women, (4) hypertension (blood pressure ≥130/85 mmHg or taking antihypertensive medication), and (5) hyperglycemia (fasting plasma glucose (FPG) ≥100 mg/dL or taking antidiabetic medication).

The WHO Regional Office for the Asia Pacific Region recommends defining obesity in Asians as those with a BMI of ≥25 kg/m^2^. The Korean Society for the Study of Obesity also studied the cutoff of BMI for obesity-related disease [22] and adopted the WHO-recommended definition. Now, Korean government organizations officially use this definition when defining and implementing health policies regarding obesity in Korea.

Subjects taking thyroid hormone or anti-obesity medicine, which can affect weight, insulin-dependent diabetes, patients with liver function abnormality (liver somatic index >3 times the normal maximum level) or renal function impairment (creatinine level >1.5 times the normal maximum level), pregnant women, and inpatients were excluded from this study.

Subjects were recruited through installing institutional review board- (IRB-) approved banners, posters, and leaflets in the hospital lobby. As an incentive for the registered test participants (both intervention and comparison groups), all expenses for medical treatment, medicine, transportation, and communication (mobile phones) were provided from the national project budget.

Eligible participants were assigned to the 2 groups with equal probability according to a randomization code. The randomization code was prepared by a block randomization method stratified (according to the enrolling clinical centre) by a statistician in a clinical trial centre (C&R Research, Seoul, South Korea). This study was an open labelled trial, blinding was not done.

The Institutional Review Board of Seoul National University Hospital approved this study (IRB number: h-1009-095-333).

### Intervention Group

**Figure 1:**
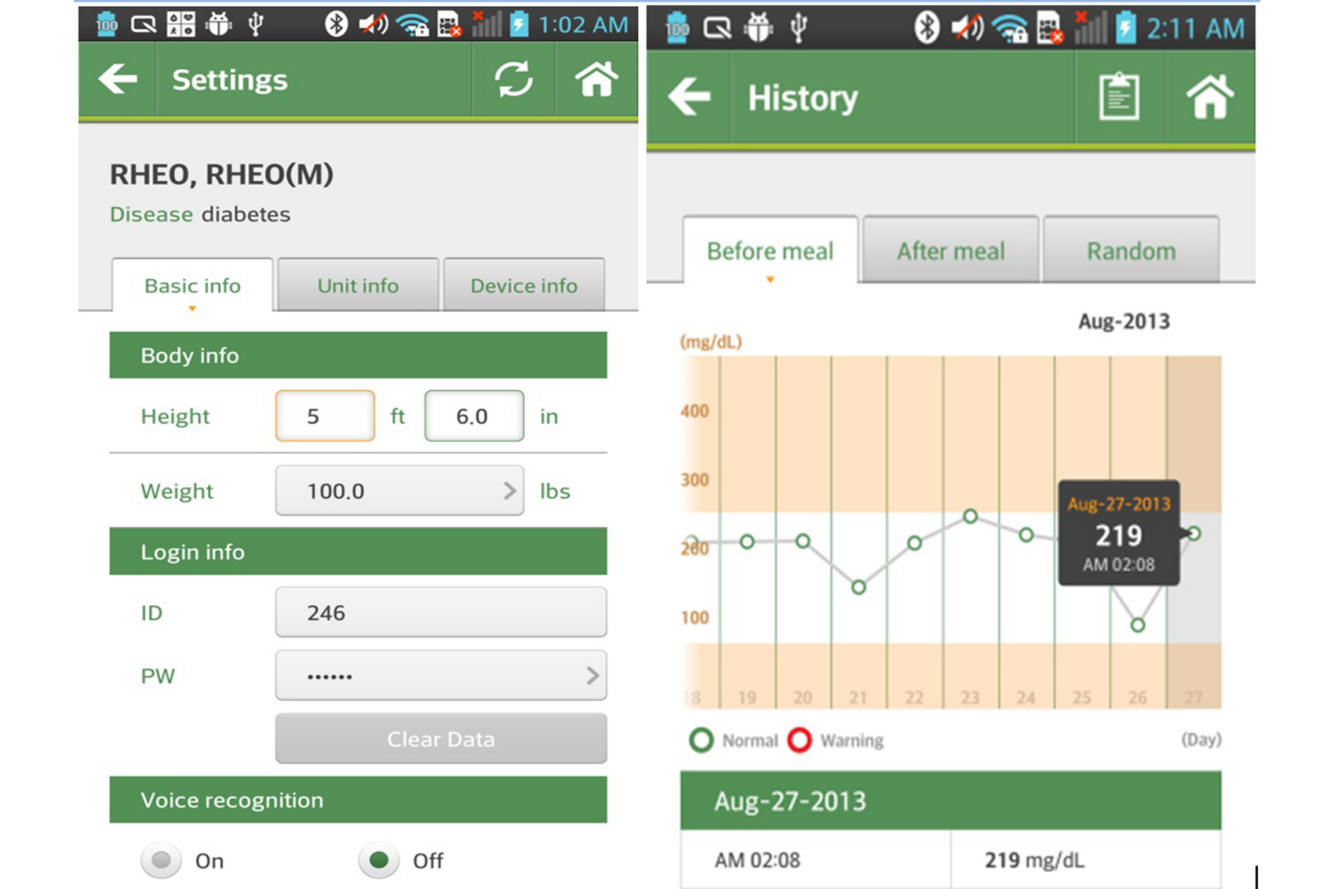
Examples of the SmartCare app.

Mobile phones for remote monitoring, body composition monitors (InBody IH-U070B) and pedometers were provided to the subjects assigned to the intervention group. Each subject measured his or her own body weight and body composition using the provided body composition monitor at the same time every day if possible (a minimum of 3 times per week), and before breakfast after waking up. After measuring the relevant values with the body composition monitor, the transmission terminal (Bluetooth) of the remote monitoring device, juxtaposed near the transmission terminal of the mobile phone transmitted the measurement data to the central server in the SmartCare center via a wireless network. Each subject carried a pedometer from the time they woke up until they went to bed. The activity level, indicated as the number of steps taken, was checked at the same time every day, if possible, and entered into the mobile phone (inputting before bed was recommended). Then, the entered data were automatically transmitted to the central server in the SmartCare center. Physicians or healthcare personnel at the SmartCare center could retrieve the hospital admission information, treatment records, name of diagnosed diseases, diagnostic examinations and functional test results, and prescription information of the test subjects by connecting to the hospital information system with the consent of the subjects. The central server in the SmartCare center transmitted the feedback based on the measured body weight and body composition to the mobile phones of the subjects according to the algorithm of the clinical decision support system (CDSS). The subjects were able to immediately check the interpretations and recommendations based on their measured values through their mobile phones (Figure 1). The educated consultants (nurse, exercise prescriber, and clinical dietitian) in the SmartCare center provided various health consultations through the patients’ telephone inquiries concerning disease management, health education, recommended exercise, medication, and proper nutrition. Also, monthly and weekly health reports based on the individual patient’s measured values and life habit records were sent directly to the patients through the SmartCare system.

### Comparison Group

Body weight scales and pedometers were provided to the subjects assigned to the control group. Body weight journals were distributed to the subjects, and each subject self-measured and recorded his/her daily weight and waist size (a minimum of 3 times per week) at the same time (before breakfast) using waist circumference. Also, they wore a pedometer during daily activities, which started from the time of waking up in the morning until bedtime in the evening. They were instructed to check and record their daily walking amount on the record sheet just before sleep.

Additionally, the subjects in the control group visited the hospitals on the same schedule as that of the intervention group and received anthropometry, consultations with physicians, and information about their nutrition and exercise.

### Study Design

The subjects who met the exclusion criteria and were excluded from the trial included diabetic patients receiving treatment, patients with diseases that might affect body weight, or those who continued to take prescribed medications. The selected subjects were randomized into a control group that received basic information on increasing physical activity and controlling diet habits, or an intervention group that received remote monitoring and uHealth care service (SmartCare) in addition to the existing treatment. Pedometers were given to all of the patients. Additionally, mobile phones and body composition monitors were provided to the intervention group, and body weight scales were provided to the control group.

The equipment (mobile phones, weight scales, and pedometers) were provided to the patients for free through the fund for the national project.

**Figure 2:**
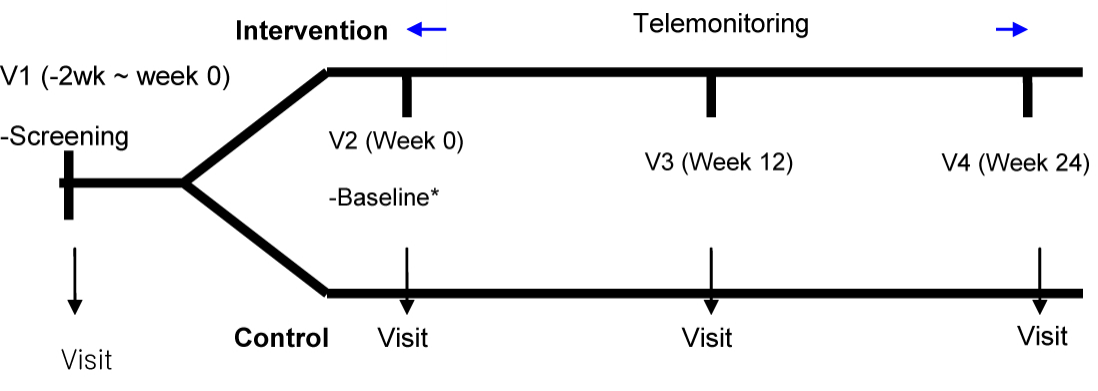
Study flowchart and design.

The subjects were asked to visit the hospitals 4 times during the 24-week period. Except when screening was performed, their body weight, body composition, and blood pressure were checked. A hematology test was performed and changes in their life habits (eg, diet intake and physical activity) were checked 3 times during the test period; once on the date the subjects were randomized, once in week 12, and once in week 24 (Figure 2).

Analysis consisted of 2 group sets: intention-to-treat (ITT) and per protocol (PP). The ITT set included all of the subjects who were enrolled in this clinical trial and were randomized. When analyzing efficacy, they were included in the treatment group into which they were randomized, regardless of the actual treatment they received. Among the subjects included in the ITT set, those who completed this clinical trial without material breach of the protocol were included in the PP set.

### Measurement

When screening was performed, the demographic information (age, gender, smoking, drinking, and others), medical history, and medication history of the subjects were investigated and recorded. Additionally, an electrocardiogram (ECG) was performed at the screening visit after resting for at least 5 minutes. When clinically significant test results were observed, the investigator determined whether to enroll the subject in the experiment.

The laboratory tests were conducted at screening, baseline, week 12, and week 24, and the tests included alanine aminotransferase (ALT), aspartate aminotransferase (AST), creatinine, lipid profile (total cholesterol, HDL-C, and TG), fasting blood sugar (FBS), and baseline glycosylated hemoglobin (HbA1c). However, ALT, AST, and creatinine tests were performed only at screening to determine trial eligibility. Lipid profiles and blood glucose tests were performed after fasting.

Weight change was the primary outcome and was evaluated with percent body fat at baseline, week 12, and week 24 and measured by nurses using a portable bioelectrical impedance analysis device (InBody U20, Seoul, Korea).

The level of physical activity was assessed and categorized using the International Physical Activity Questionnaire (IPAQ) at baseline, week 12, and week 24. The amount of physical activity each week was calculated with a continuous variable, the Metabolic Equivalent of Task (MET) using the following equation:

Total MET min/week = [walking METs×min×days] + [moderate METs×min×days] + [vigorous METs×min×days])

Concerning the method for measuring the caloric intake variables, daily meal record cards (3-day recall dietary assessment) were distributed to the subjects during their initial visit and the subjects were instructed to write their own 3-day meal record just before the baseline visit, the next visit after 12weeks, and the final visit after 24weeks. The self-completed daily meal records were collected from the subjects during their final visit and the dietitian performed the calorie calculation using the nutrient evaluation program CAN-Pro 3.0 software (The Korean Nutrition Society, 2006) [23].

### Statistical Analysis

Each group initially consisted of 167 subjects chosen using a 5% significance level, 90% power, and estimating the mean difference in weight change between the 2 groups to be 1.81 kg (SD 4.81 and 5.36 kg). Considering a 25% drop out rate, the final sample size consisted of 223 subjects for each group (N=446 subjects) [24-27].

Descriptive statistics including the number of observed subjects, mean, and median (range) of body weight measured at baseline, week 12, and week 24, and the changes in measured values at week 24 compared to baseline were presented for each group. To identify the difference between the groups with respect to body weight changes at week 24 compared to the baseline, analysis of covariance (ANCOVA), including the clinical trial institution and the body weight at baseline as covariates, was performed.

For continuous data, such as changes in BMI, body fat percentage, waist measurement, lipid profile, blood pressure, the number of metabolic syndrome elements, diet intake (kcal), physical activity, number of steps taken, and weight to measure physical activity, descriptive statistics including the number of observed subjects, mean, and median (range) were presented for each group. To identify the difference between the groups at week 24 compared to the baseline, ANCOVA or rank transformation ANCOVA was performed. When conducting ANCOVA, the clinical trial institution and the baseline values of relevant parameters were set as covariates.

For categorical data including the ratio of the subjects whose body weights decreased by ≥10%, the changes in the prevalence rates of metabolic syndrome and the changes in life habits (ie, smoking and drinking, the frequency, rates, and 95% confidence interval were presented for each group. To identify the difference in rates between the groups at weeks 12 and 24, the Cochran-Mantel-Hansel (CMH) test was performed using the clinical trial institution as a covariate.

To identify the satisfaction level of the subjects (only for intervention group), the scores of the satisfaction survey items related to the usage convenience of the devices were measured at weeks 12 and 24, and descriptive statistics including the number of observed subjects, mean, and median (range) on the measured scores were presented.

The adverse events occurring after randomization were collected and analyzed. The frequency, percentage, and 95% confidence intervals of the adverse events and serious adverse events were presented. To find the difference between the groups in the frequency of adverse and serious adverse events, Pearson’s chi-square test or Fisher’s exact test was performed. The adverse and serious adverse events were coded according to the Medical Dictionary for Regulatory Activities (MedDRA) system organ class (SOC) and preferred term (PT), and the number of types, frequency, and the number of cases of the coded adverse events were presented. Additionally, the numbers of adverse events occurrences and percentages were presented by severity, and a detailed statement on the serious adverse events was presented.

All analyses were conducted using STATA version 12.1 (StatCorp, Houston, TX) for Windows software. *P* values less than .05 were considered statistically significant.

##  Results

### Subject Participation

**Figure 3:**
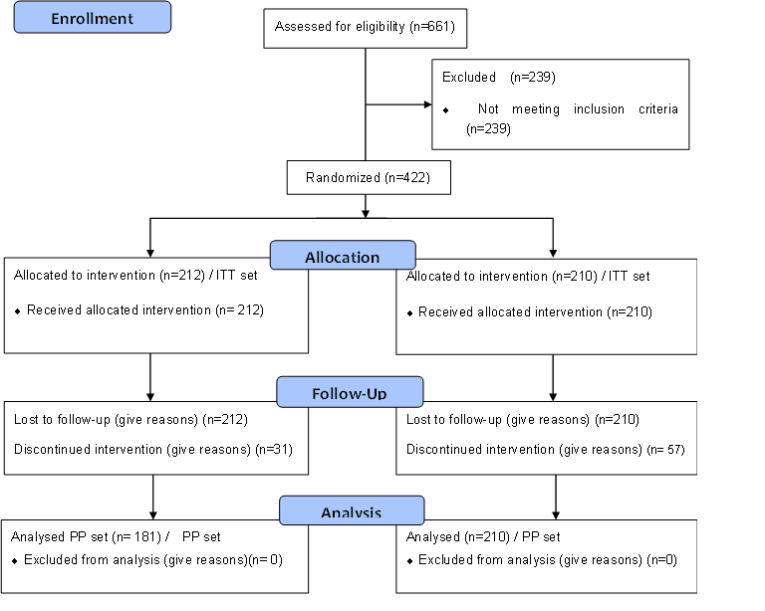
Selection of the study participants.

During the clinical trial, a total of 661 subjects went through screening, and 442 of them were identified as subjects with metabolic syndrome. Subjects were randomized into the intervention (N=212) or control (N=210) group. Therefore, the ITT set included a total of 422 subjects. Because a total of 31 (14.6%, 31/212) subjects dropped out of the intervention group during the observation period of 24 weeks, 181 (85.3%, 181/212) subjects completed the trial. A total of 57 (27.1%, 57/210) subjects dropped out of the control group, and 153 (72.9%, 153/210) subjects completed the trial. Thus, the PP set included a total of 334 subjects (Figure 3).

### Demographic Data and Characteristics of Subjects Prior to Treatment

The conditions of the subjects before providing medical service were compared between groups, including the demographic information of the subjects included in the ITT set. The mean ages were 46.78 years (SD 13.11) for the intervention group and 50.35 years (SD 14.24) for the control group, indicating a statistically significant difference in the age distribution between the groups (*P*=.008). However, there was no significant difference between the 2 in terms of age groups (*P*=.269). The number of male and female subjects in the intervention group were 113 (53.3%, 113/212and 99 (46.7%, 99/212), respectively. In the control group, the number of male subjects was 102 (48.6%, 102/210) and the number of female subjects was 108 (51.4%, 108/210) demonstrating no statistically significant difference in gender distribution between the groups (*P*=.331). The mean BMI was 29.42 kg/m^2^ (SD 3.53) for the intervention group and 29.40kg/m^2^ (SD 3.39) for the control group, indicating no statistically significant difference in the distribution of BMI between the groups (*P*=.934).

With respect to the education level of subjects in the 2 groups, 136 (64.2%, 136/212) subjects in the intervention group were “college graduates or higher”, whereas 109 (51.9%, 109/210) in the control group were “college graduates or higher”. Statistically significant differences in education levels were observed among the “elementary school graduates” and “college graduates or higher” (*P*=.001). Moreover, body weight (*P*=.343), height (*P*=.131), smoking (*P*=.475), and drinking (*P*=.726) were not statistically significantly different between the 2 groups (Multimedia Appendix 1).

### Efficacy Evaluations

#### Primary Efficacy Evaluation

The body weights at baseline, week 12, and week 24 were summarized in the descriptive statistics, and the body weight changes at week 24 compared to baseline were assessed. In the ITT set, the mean body weights of the intervention group at baseline and week 24 were 81.13 kg (SD 14.77) and 77.87 kg (SD 13.99), respectively. The mean body weight at week 24 decreased by 2.21 kg (SD 3.60) compared to the weight at baseline, which was statistically significant (*P*<.001). The mean body weights of the control group at baseline and week 24 were 79.74 kg (SD 15.28) and 77.02 kg (SD 13.33), respectively. The mean body weight at week 24 decreased by 0.77 kg (SD 2.77) compared to baseline, which was statistically significant (*P*<.001). In the comparison between the groups, the mean body weight change of the intervention group at week 24 compared to baseline was significantly higher than that of the control group (*P*<.001) (Table 1).

In the PP set, the mean body weights of the intervention group at baseline and week 24 were 79.93 kg (SD 13.55) and 77.64 kg (SD 13.91), respectively. The mean body weight at week 24 decreased by 2.29 kg (SD 3.62) compared to baseline, which was statistically significant (*P*<.001). The mean body weights of the control group at baseline and week 24 were 77.89 kg (SD 13.68) and 77.02 kg (SD 13.28), respectively. The mean body weight at week 24 decreased by 0.86 kg (SD 2.84) compared to baseline, which was statistically significant (*P*<.001). As in the ITT set, the mean body weight change of the intervention group at week 24 compared to baseline was significantly higher than that of the control group (*P*<.001) (Table 2).

**Table 1:** Changes in weight (baseline versus 24 weeks) in the ITT set.

Weight, kg		Intervention group	Control group	Between groups *P* value
**Baseline**				
	n	212	209	
	Mean (SD)	81.13 (14.77)	79.74 (15.28)	
	Median	79.85	76.90	
	Min, max	55.20, 144.00	54.00, 141.10	
**Week 12**				
	n	196	179	
	Mean (SD)	77.85 (13.46)	76.67 (13.26)	
	Median	76.85	74.10	
	Min, max	49.80, 135.70	53.40, 118.10	
**Week 24**				
	n	196	181	
	Mean (SD)	77.87 (13.99)	77.02 (13.33)	
	Median	77.35	75.10	
	Min, max	50.10, 141.00	53.20, 117.90	
**Change W24** ^a^				
	n	196	181	
	Mean (SD)	-2.21 (3.60)	-0.77 (2.77)	<.001^b^
	Median	-2.10	-0.70	
	Min, max	-17.00, 5.60	-20.40, 6.60	
Within group, *P* value		<.001^c^	<.001^c^	

^a^Change W24=week 24−baseline

^b^ANCOVA using the site and baseline weight as covariates

^c^Paired *t* test

**Table 2:** Changes in weight (baseline versus 24 weeks) in the PP set.

Weight, kg		Intervention group	Control group	Between groups *P* value
**Baseline**				
	n	181	153	
	Mean (SD)	79.93 (13.55)	77.89 (13.68)	
	Median	79.10	74.70	
	Min, max	55.20, 135.40	54.00, 120.10	
**Week 12**				
	n	181	152	
	Mean (SD)	77.62 (13.38)	76.61 (13.21)	
	Median	76.80	74.00	
	Min, max	49.80, 135.70	53.40, 118.10	
**Week 24**				
	n	181	153	
	Mean (SD)	77.64 (13.91)	77.02 (13.28)	
	Median	76.80	75.00	
	Min, max	50.10, 141.00	53.20, 117.90	
**Change W24** ^a^				
	n	181	153	
	Mean (SD)	-2.29 (3.62)	-0.86 (2.84)	<.001^b^
	Median	-2.10	-0.80	
	Min, max	-17.00, 5.60	-20.40, 6.60	
Within group *P* value		<.001^c^	<.001^c^	

^a^Change W24=week 24−baseline

^b^ANCOVA using the site and baseline weight as covariates

^c^Paired *t* test

#### Secondary Efficacy Evaluation

Among the secondary efficacy evaluation parameters, BMI, rate of body fat, decrement of waist measurement, and diet habit improvement from baseline to week 24 were superior in the intervention group compared with the control group (body fat rate *P*=.001, diet habit *P*=.012, and others *P*<.001). In particular, the mean BMIs of the intervention group in the ITT set at baseline and week 24 were 29.42 kg/m^2^ (SD 3.53) and 28.33 kg/m^2^ (SD 3.46), respectively. The mean BMI at week 24 decreased by 0.83 kg/m^2^ (SD 1.31) compared to baseline, which was statistically significant (*P*<.001). The mean BMIs of the control group at baseline and week 24 were 29.40 kg/m^2^ (SD 3.39) and 28.74 kg/m^2^ (SD 2.88), respectively. The mean BMI at week 24 decreased by 0.28 kg/m^2^ (SD 1.03) compared to baseline, which was statistically significant (*P*=<.001). In the comparison between the groups, the change in BMIs of the intervention group at week 24 compared to baseline was significantly higher than that of the control group (*P*<.001) (Table 3).

A similar trend was observed in the PP set (Table 4). However, the ratio of the patients whose body weight decreased by ≥10% and the lipid profile, blood pressure, prevalence of metabolic syndrome, change in the number of metabolic syndrome elements, smoking rate, drinking rate, and physical activity were not statistically significantly different between the groups.

**Table 3:** Changes in BMI (baseline versus 24 weeks) in the ITT set.

BMI, kg/m^2^		Intervention group	Control group	Between groups *P* value
**Baseline**				
	n	212	209	
	Mean (SD)	29.42 (3.53)	29.40 (3.39)	
	Median	28.70	28.90	
	Min, max	24.90, 46.80	24.90, 41.80	
**Week 12**				
	n	196	179	
	Mean (SD)	28.35 (3.25)	28.59 (2.84)	
	Median	27.85	28.10	
	Min, max	22.10, 40.10	24.00, 38.90	
**Week 24**				
	n	196	181	
	Mean (SD)	28.33 (3.46)	28.74 (2.88)	
	Median	27.55	28.20	
	Min, max	22.60, 41.40	23.90, 39.70	
**Change W24** ^a^				
	n	196	181	
	Mean (SD)	-0.83 (1.31)	-0.28 (1.03)	<.001^b^
	Median	-0.80	-0.20	
	Min, max	-5.80, 2.20	-6.70, 2.40	
Within the group *P* value		<.001^c^	<.001^c^	

^a^Change W24=week 24−baseline

^b^ANCOVA using the site and baseline weight as covariates

^c^Paired *t* test

**Table 4:** Changes in BMI (baseline versus 24 weeks) in the PP set.

BMI, kg/m^2^		Intervention group	Control group	Between group*s P* value
**Baseline**				
	n	181	153	
	Mean (SD)	29.18 (3.13)	29.08 (2.90)	
	Median	28.60	28.80	
	Min, max	25.00, 41.40	25.00, 39.70	
**Week 12**				
	n	181	152	
	Mean (SD)	28.34 (3.23)	28.60 (2.76)	
	Median	27.80	28.10	
	Min, max	22.10, 40.10	24.00, 38.50	
**Week 24**				
	n	181	153	
	Mean (SD)	28.32 (3.44)	28.74 (2.81)	
	Median	27.50	28.30	
	Min, max	22.60, 41.40	23.90, 39.70	
**Change W24** ^a^				
	n	181	153	
	Mean (SD)	-0.86 (1.32)	-0.33 (1.04)	<.001^b^
	Median	-0.80	-0.30	
	Min, max	-5.80, 2.20	-6.70, 2.40	
Within the group *p*-value		<.001^c^	<.001^c^	

^a^Change W24=week 24−baseline

^b^ANCOVA using the site and baseline weight as covariates

^c^Paired *t* test

### Subject Satisfaction

The convenience of device usage, satisfaction with the SmartCare center service, and overall satisfaction of the remote monitoring were determined at weeks 12 and 24, based on a 5-point scale where 5 corresponded to highly satisfied. At week 12, the convenience of device usage, satisfaction with the SmartCare center service, and overall satisfaction of the remote monitoring were found to be 3.54 (SD 1.02), 4.08 (SD 0.86), and 3.93 (SD 0.86), respectively. At week 24, the satisfaction with the convenience of device usage was 3.52 (SD 0.99), SmartCare center service was 4.14 (SD 0.88), and overall satisfaction of the remote monitoring was 3.92 (SD 0.85).

### Safety Results

The rates of adverse events in the intervention group and control group were 14.2% (30/212, 43 cases) and 13.3% (28/210, 40 cases), respectively. The rates of serious adverse events in the intervention group and control group were 1.4% (3/212, 3 cases) and 2.4% (5/210, 5 cases), respectively. Due to serious adverse events, the intervention group showed 1 case of ankle fracture, and the control group showed 1 case of dislocated vertebra, stress urinary incontinence, and knee operation.

After the physical examination, no subject in either of the 2 groups had abnormalities at week 24 after showing no abnormalities at baseline.

Pulse reduction at week 24 compared to baseline was 2.84 beats/min (SD 10.01) for the intervention group and 0.94 beats/min (SD 8.47) for the control group. The difference between the groups was statistically significant (*P*=.049), but such a change is considered not to be directly related to SmartCare.

##  Discussion

### Principal Findings

To the best of our knowledge, this study was the first domestic government project to estimate the usefulness of SmartCare in managing chronic disease. Weight decrement after 24 weeks from the baseline, a primary efficacy evaluation parameter, was 2.21 kg (SD 3.60) for the intervention group and 0.77 kg (SD 2.77) for the control group, and the intervention group showed a higher rate of reduction compared to the other group (*P*<.001). Among the secondary efficacy evaluation parameters, BMI, body fat rate, decrement of waist measurement, and diet habit improvement ratings after 24 weeks were superior in the intervention group (body fat rate *P*<.001, diet habit *P*=0.012, and others *P*<.001). The proportion of patients whose body weight decreased by ≥10%, the lipid profiles, blood pressure, prevalence of metabolic syndrome, change in the number of metabolic syndrome elements, smoking rate, drinking rate, and physical activity were not significantly different.

uHealth care, an abbreviation for ubiquitous health care, refers to a health care medical service in which information and communication technologies are combined with medicine so that patients can be provided with prevention, diagnosis, treatment, and follow-up services anytime and anywhere, even if they do not directly visit hospitals [28,29]. uHealth care is a medical service developed to collect real-time health-related information without limits pertaining to time or location, and to perform continuous monitoring and treatment to examine health conditions in advance and prevent diseases, rather than solely providing treatment after the onset of disease. Because of the sudden increase in medical costs due to population aging and the increase of patients with chronic diseases in modern society, the need for the development of such a service is increasing, as is the need to build a cost-effective medical system and improve the quality of health and medical treatment services [30].

This clinical trial was designed in consideration of such situations. Here, we compared the SmartCare service (uHealth care) with the existing treatment to evaluate the effect of the service on body weight, as well as its safety in obese patients who need constant monitoring.

Overweight and obesity due to a Westernized diet and lack of exercise are serious problems all over the world and have adverse effects not only on personal health but also on national economies. To solve these problems, an approach including prevention should be taken, rather than relying only on medical treatment after the occurrence of disease. In addition, long-term treatments such as improving individual life habits through continuous care are paramount [31].

Metabolic syndrome is a disease caused by insulin resistance, which is usually linked to overweight and obesity. Thus, weight control is vital for this condition. To lose weight, exercise and a controlled diet are absolutely important [5,11,32,33]. Practicing diet therapy can cut down on fat as well as lean body mass. This drops the basic metabolic rate, easily causing the yo-yo effect where the patient gains weight even from small food intake.

### Comparison With Prior Work

Various attempts to prevent obesity have been made, including a case where healthy eating habits and adequate exercise to maintain reduced weight were carried out in a community setting [34,35]. Cases where competition among members to maintain healthy lifestyles and to lose weight was encouraged with the use of aggressive measures have been reported in a timely fashion with the introduction of telemedicine [9]. However, the exchange of food and exercise information through the Internet was found to be more effective in weight loss and maintenance than traditional methods of self-maintenance and self-management [36].

This clinical trial paired the SmartCare service together with the existing treatment (intervention group) to only the existing treatment (control group) and compared the mean change in body weight at week 24 to baseline between the 2 groups. The test results showed significantly higher changes in body weight in the intervention group than in the control group, proving that the SmartCare service shows higher efficacy when combined with the existing treatment than when only the existing treatment was provided. Additionally, we obtained results consistent with the primary efficacy evaluation in BMI, body fat rate, and waist measurements, which are directly related to obesity and body weight. In the satisfaction survey for the SmartCare service, convenience of device usage, satisfaction with the SmartCare Center service, and overall satisfaction with remote monitoring, all received responses of were 'satisfactory' or 'very satisfactory' from ≥50% of the users. Studies conducted in other countries have found similar results. These studies showed that various methods implemented to motivate patients to increase physical activity, control their diets, and maintain healthy life habits mostly produced positive results [16,20,37-44].

### Study Strengths and Limitations

Based on the number of times information was entered on the mobile phone for diet intake and the amount of exercise, the subgroup analysis of this study revealed that the scope of weight loss was considerably increased. Through this revelation, the level of interest in using uHealth in real-time was found to be an important variable that can heavily influence the level of weight loss. The difference between this study compared to previous studies from abroad is that the mean age of the subjects was between the mid-40s to the early 50s, older than the ages of the subjects included in the other studies. Thus, this study is meaningful in that it showed that the concept of uHealth is not the exclusive property of younger generations who use advanced devices. It showed that anyone who uses a mobile phone in modern society could use uHealth accordingly.

However, the fact that highly educated young individuals were assigned to the intervention group, despite the random selection, may work as a selection bias when evaluating the effects of SmartCare. Nonetheless, this research also revealed that the number of people who failed in the control group was twice that of the intervention group, thereby suggesting the possibility that SmartCare increased participants' adherence to a weight loss program compared to weight loss means using traditional methods.

On the other hand, the rates of the subjects whose body weight decreased by ≥10%, lipid profiles (total cholesterol, HDL-C, low-density lipoprotein cholesterol (LDL-C), and TG), changes in systolic and diastolic blood pressure, rate of patients with metabolic syndrome, change in the number of metabolic syndrome elements, smoking rate, and drinking rate were not statistically significantly different between the intervention and control groups. There are a few possible causes for this observation. First, subjects with reduced weight during the initial phase of the study showed a tendency to have slight increases in weight towards the latter phase of the study. Second, it is difficult to confirm whether eating habits, specifically the control of calories and salt intake, improved compared to the increased physical activity and weight loss effect. The risk factors of metabolic syndrome are expected to decrease when the effects of weight reduction are sustained for a long period of time, and such benefits can be confirmed with a longer research plan for weight maintenance. Furthermore, if the reduction can be quantitatively proved through feedback regarding the controlled salt and total calorie intake to improve eating habits, it may be possible to observe the number of metabolic syndrome factors, such as blood pressure and the change in the lipid profile, of the participants through subgroup analysis.

### Conclusions

Through analyzing obesity evaluation indexes such as BMI and including weight, body fat rate, and waist measurements, we found that the SmartCare service is an effective way to control the weight of obese patients with metabolic syndrome.

The positive effects of the development of uHealth on the medical field can influence not only health care providers but also various fields, including health care centers and network operators, can provide medical service anytime and anywhere without patients having to visit hospitals during operating hours, and can provide individually customized service for health improvement and disease prevention for identical conditions in contrast to the usual patient-doctor relationship [45].

## Multimedia Appendix 1

Demographic characteristics of the patients at baseline in the ITT set.

## Abbreviations

ALT: alanine aminotransferase
ANCOVA: analysis of covariance
AST: aspartate aminotransferase
BMI: body mass index
FBS: fasting blood sugar
FPG: fasting plasma glucose
HbA1c: baseline glycosylated hemoglobin
HDL-C: high-density lipoprotein cholesterol
IRB: institutional review board
ITT: intention-to-treat
MET: Metabolic Equivalent of Task
PP: per protocol
TG: triglyceride
uHealth: ubiquitous health
WHO: Who Health Organization
