# Supplementary material for: The Effectiveness of Mobile Phone-Based Care for Weight Control in Metabolic Syndrome Patients: Randomized Controlled Trial
Source: JMIR Mhealth Uhealth. 2015 Aug 20;3(3):e83. doi: 10.2196/mhealth.4222 (PMC4705013; doi:10.2196/mhealth.4222)
Supplement: Supplementary file 1 [file mhealth_v3i3e83_app1.pdf]

Characteristic		Intervention group, N=212	Control group, N=210	*P* value
**Age**				
	n	212	210	
	Mean (SD)	46.78 (13.11)	50.35 (14.24)	.0077[a]
	Median	48.00	52.00	
	Min, max	20.00, 72.00	21.00, 82.00	
	20-29 years, n (%)	23 (10.9)	18 (8.6)	.2691[b]
	30-39 years, n (%)	48 (22.6)	40 (19.1)	
	40-49 years, n (%)	42 (19.8)	34 (16.2)	
	50-59 years, n (%)	59 (27.8)	61 (29.1)	
	≥60 years, n (%)	40 (18.9)	57 (27.1)	
**Sex**				
	n	212	210	
	Male, n (%)	113 (53.3)	102 (48.6)	.3311[b]
	Female, n (%)	99 (46.7)	108 (51.4)	
**BMI, kg/m^2**				
	n	212	209	
	Mean (SD)	29.42 (3.53)	29.40 (3.39)	.9342[a]
	Median	28.70	28.90	
	Min, max	24.90, 46.80	24.90, 41.80	
**Weight, kg**				
	n	212	209	
	Mean (SD)	81.13 (14.77)	79.74 (15.28)	.3427[a]
	Median	79.85	76.90	
	Min, max	55.20, 144.00	54.00, 14.10	
**Height, cm**				
	n	212	209	
	Mean (SD)	165.58 (9.18)	164.12 (10.61)	.1310[a]
	Median	166.40	163.00	
	Min, max	144.50, 188.90	142.80, 189.00	
**Smoking, n (%)**				
	n	211	203	
	Non-smoker	127 (60.2)	129 (63.6)	.4753[b]

	Former-smoker	52 (24.6)	40 (19.7)	
	Smoker	32 (15.2)	34 (16.8)	
**Drinking, n (%)**				
	n	211	206	
	Non-drinker	78 (37.0)	84 (10.8)	.7258[b]
	Former-drinker	16 (7.6)	15 (7.3)	
	Drinker	117 (55.5)	107 (51.9)	
**Education, n (%)**				
	n	212	210	
	Uneducated	0 (0.0)	0 (0.0)	.0011[b]
	Primary school graduate	4 (1.9)	21 (10.0)	
	Middle school graduate	9 (4.3)	15 (7.1)	
	High school graduate	63 (29.7)	65 (31.0)	
	College graduate or higher	136 (61.2)	109 (51.9)	

[a]Two-sample *t* test

[b]Pearson's chi-square test
